# Medial open‐wedge high tibial osteotomy demonstrates an 85.9% non‐conversion rate and 76.8% patient satisfaction at a minimum follow‐up of 10 years

**DOI:** 10.1002/jeo2.70841

**Published:** 2026-07-07

**Authors:** Joachim‐Artus Junghans, Martin Häner, Bernd Wolfarth, Wolf Petersen

**Affiliations:** ^1^ Department of Orthopaedics and Trauma Surgery Martin Luther Hospital Berlin Germany; ^2^ Department of Sports Medicine, Charité—Universitätsmedizin Berlin Freie Universität Berlin and Humboldt‐Universität zu Berlin Berlin Germany

**Keywords:** joint preservation, long‐term outcomes, medial opening wedge high tibial osteotomy, survival, varus deformity

## Abstract

**Purpose:**

To evaluate long‐term survival and functional outcomes following medial open wedge high tibial osteotomy (MOWHTO) using a locking plate.

**Methods:**

One hundred fifty‐four patients underwent MOWHTO for isolated medial compartment osteoarthritis at Martin Luther Hospital, Berlin, Germany. After application of the predefined inclusion and exclusion criteria, 26 patients were excluded, leaving 126 eligible patients. Of these, two patients had died, and 18 were lost to follow‐up. The primary endpoint was survival, defined as the absence of conversion to total knee arthroplasty (TKA). Secondary outcomes included patient‐reported outcome measures (Knee Injury and Osteoarthritis Outcome Score [KOOS]), pain, satisfaction, sporting activity (Tegner scale) and complications. Kaplan–Meier analysis was performed for survival, and potential risk factors for conversion were analysed. The mean follow‐up was 13.3 years (range, 10.2–18.3).

**Results:**

A total of 99 patients were assessed at a mean follow‐up of 13.3 years (range, 10.2–18.3), comprising 35.4% women and 64.6% men. The mean age was 48.4 ± 8.3 years at the time of surgery and 61 ± 8 years at final follow‐up. The cumulative non‐conversion rate was 91.9% at 5 years, 85.9% at 10 years and 80.8% at final follow‐up (13.3 years). Patients in the conversion group were significantly older (64.89 ± 8.0 years) than those in the non‐conversion group (60.0 ± 7.6 years, *p* = 0.017). Female sex (*p* = 0.287), body mass index (BMI, *p* = 0.404), smoking (*p* = 0.647), patellofemoral cartilage damage (*p* = 0.254) and lateral cartilage damage (*p* = 0.492) were not significantly associated with conversion. KOOS subscales at follow‐up reached or exceeded published Patient Acceptable Symptom State thresholds. Mean pain scores were 1.4 at rest and 2.7 during walking. Overall satisfaction was 76.8%, with significantly higher satisfaction among patients without conversion (83.8%) compared to those with conversion (47.4%, *p* = 0.002). Sports participation decreased significantly, with Tegner scores declining from 4.7 to 3.8, although most patients remained physically active (*p* = 0.001). The overall complication rate was 12.1%, with overcorrection being the most common event.

**Conclusion:**

MOWHTO with angular‐stable fixation demonstrated high long‐term survival, favourable functional outcomes and high patient satisfaction. Patients in the conversion group were significantly older than those in the non‐conversion group. These findings suggest that MOWHTO is a safe and effective joint‐preserving procedure in appropriately selected patients with varus malalignment and medial compartment osteoarthritis.

**Level of Evidence:**

Level IV.

**Abbreviations**
BMI: body mass index
CI: confidence interval
HTO: high tibial osteotomy
KOOS: Knee Injury and Osteoarthritis Outcome Score
MOWHTO: medial opening wedge high tibial osteotomy
NRS: numerical rating scale
PASS: Patient Acceptable Symptom State
PF: patellofemoral
PROMs: patient‐reported outcome measures
TKA: total knee arthroplasty

## INTRODUCTION

Medial opening‐wedge high tibial osteotomy (MOWHTO) is a well‐established method to address varus malalignment, medial compartment osteoarthritis and related pathologies of the knee [23, 37]. Correction of the leg axis has gained popularity due to its ability to restore joint biomechanics and alleviate symptoms associated with medial compartment overload [23, 27, 34, 37, 38].

Two main surgical strategies exist for correcting tibial varus deformity: lateral closing‐wedge and medial opening‐wedge osteotomy [7, 35]. A longstanding limitation of the medial opening technique was the difficulty of adequately stabilizing the osteotomy gap with conventional plates. The introduction of angular‐stable fixation systems has overcome this problem and has contributed to a renewed preference for this method [37]. In contrast, the lateral closing‐wedge technique carries a risk of peroneal nerve injury [36].

The TomoFix™ plate (DePuy Synthes) was the first osteosynthesis system specifically developed for MOWHTO, as described by Staubli et al. [37]. Its high primary stability allows early mobilization without the need for bone grafting. While short‐ and mid‐term results of this technique have been consistently favourable [27, 31], only a limited number of studies have reported on long‐term survival [8, 17, 25, 33, 34]. These reports demonstrated 10‐year survival rates ranging from 67% to 95%, but due to small sample sizes, reliable identification of risk factors for conversion to total knee arthroplasty (TKA) has remained difficult.

The primary aim of this study was to determine the long‐term non‐conversion rate to TKA after MOWHTO stabilized with a locking plate. We hypothesized that the non‐conversion rate at a minimum follow‐up of 10 years would exceed 80%.

The predefined threshold of 80% was based on previously published long‐term studies reporting non‐conversion or survivorship rates of approximately 80% or higher at 10 years after MOWHTO in appropriately selected patients [17, 33]. Hantes et al. demonstrated durable functional and radiological outcomes at long‐term follow‐up in younger patients (95% non‐conversion rate), while Schuster et al. reported favourable 10‐year results even in patients with advanced medial osteoarthritis (non‐conversion rate 81.7%). Therefore, 80% was chosen as a conservative and clinically meaningful benchmark for long‐term joint preservation.

The secondary aim was to identify potential risk factors for conversion to TKA and to evaluate patient‐reported outcomes, including patient satisfaction. Smoking, obesity and advanced cartilage damage were hypothesized to be associated with an increased risk of conversion and inferior clinical outcomes.

## MATERIALS AND METHODS

### Study design and ethics

The study was approved by the Medical Ethics Committee of the Medical Faculty of Charité—Universitätsmedizin Berlin and conducted at the Department of Orthopaedics and Trauma Surgery, Martin Luther Hospital, Berlin, Germany. Although this study has a retrospective design, the study protocol, including predefined primary and secondary endpoints as well as statistical analyses, was prospectively registered to ensure transparency, minimize selective reporting bias and enhance methodological rigour (DRKS00037298).

### Patients

Between 2003 and 2008, 154 patients underwent MOWHTO performed by three different orthopaedic surgeons at Martin Luther Hospital, Berlin, Germany. After application of the predefined inclusion and exclusion criteria, 26 patients were excluded, leaving 126 eligible patients (Table 1). Of these, two patients had died, and 18 were lost to follow‐up.

**Table 1:** Inclusion and exclusion criteria.

Inclusion criteria	Exclusion criteria
Patient with medial open‐wedge osteotomy	Additional ACL or PCL reconstruction Follow up <10 years Insufficient German Language Skills Age <18 years

Abbreviations: ACL, anterior cruciate ligament; PCL, posterior cruciate ligament.

Informed consent was obtained in two stages. At the time of initial treatment, all patients provided written consent allowing the use of anonymized clinical data for research purposes. Prior to final follow‐up, additional written informed consent was obtained from all participating patients for study inclusion and completion of patient‐reported outcome measures (PROMs).

Written informed consent was obtained from 106 patients; three patients were excluded due to incomplete consent documentation. In four patients who underwent bilateral high tibial osteotomy (HTO), only the first operated knee was included in the analysis. Consequently, a final cohort of 99 patients was included in the study, corresponding to a follow‐up rate of 78.6% among eligible patients. Preoperative and operative data were obtained from hospital records.

### Risk factor assessment

After inclusion, hospital charts were reviewed for each included patient to obtain relevant clinical data (baseline characteristics), including age, gender, smoking status, body mass index (BMI), cartilage damage in the medial, lateral and patellofemoral (PF) compartments and complications.

Cartilage damage was assessed based on surgical reports and graded according to the International Cartilage Repair Society (ICRS) classification, which is routinely used at our institution for documenting cartilage lesions.

To assess potential attrition bias, baseline demographic and clinical characteristics (age, sex, BMI and preoperative osteoarthritis grade) of patients lost to follow‐up were compared with those included in the final analysis, based on available hospital records.

### Indication for medial opening wedge osteotomy

The indication for MOWHTO was symptomatic medial compartment osteoarthritis with varus malalignment >5°. Patients were eligible for surgery if cartilage damage in the lateral and PF compartments did not exceed ICRS grade 2.

### Surgical technique

Preoperative planning was performed according to Miniaci et al. with Fujisawa's point as a target [24]. All procedures were conducted under general or spinal anaesthesia. Perioperative antibiotic prophylaxis consisted of Cefuroxime (1.5 g), administered 30 min prior to incision. Routine arthroscopy was performed to assess cartilage status and treat intra‐articular pathology as necessary.

The osteotomy technique followed the description by Stäubli et al. [36]. Through an anteromedial approach, the superficial medial collateral ligament was released with a rasp. A Hohmann retractor protected the posterior cortex and neurovascular structures. Two 2.5‐mm guide wires were inserted under fluoroscopy, with the hinge located just above the fibular head. A biplanar osteotomy was performed using an oscillating saw cooled with sterile saline, leaving 5–10 mm of the lateral cortex intact. The gap was gradually opened with chisels and a spreader until the planned correction was achieved. Alignment was confirmed intraoperatively with a long alignment rod. Fixation was performed using an angular stable plate (TomoFix™; DePuy Synthes). No bone grafting was applied.

Postoperatively, patients were mobilized with partial weight‐bearing for 6 weeks. No range‐of‐motion restrictions were applied. Thromboprophylaxis with low‐molecular‐weight heparin was administered, and sutures were removed at 12 days.

### Follow‐up and outcome measures

The primary endpoint was survival of the HTO, defined as absence of conversion to TKA or unicompartmental knee arthroplasty (UKA). Information regarding conversion to TKA was obtained through structured telephone interviews. Hardware‐related revisions (e.g., plate removal, loss of correction or non‐union requiring reoperation) were recorded as complications but were not classified as failure unless conversion to arthroplasty occurred.

Secondary outcomes included patient satisfaction, PROMs, pain, activity level and complications. PROMs and satisfaction data were collected using standardized questionnaires distributed either electronically via email or by postal mail. Complications were assessed by reviewing the patients' medical records.

Overall satisfaction with the surgical outcome was assessed using a five‐point Likert scale (‘very satisfied’, ‘satisfied’, ‘neutral’, ‘dissatisfied’, ‘very dissatisfied’), consistent with prior orthopaedic studies on TKA and HTO [22, 40, 41]. Willingness to undergo the procedure again was assessed using a similar five‐point Likert scale (‘definitely yes’, ‘yes’, ‘neutral’, ‘no’, ‘definitely no’), modelled after standardized satisfaction instruments with demonstrated reliability and validity in orthopaedic surgery cohorts [9].

Patients completed the Knee Injury and Osteoarthritis Outcome Score (KOOS) independently after standardized instruction. Pain at rest and during walking was rated on a numerical rating scale (NRS) (0–10) [30].

Sporting activity before and after surgery was assessed and classified on the Tegner activity scale [39].

To facilitate clinical interpretation, minimal clinically important difference (MCID) thresholds reported by Collins et al. were applied for the KOOS subscales (Pain ≥16.7, Symptoms ≥10.7, Sport/Rec ≥12.5 and quality of life [QoL] ≥15.6) to distinguish statistically significant from clinically meaningful differences, while for the Tegner activity scale, a change of ≥1 point was regarded as relevant based on previously published thresholds [6, 30, 38]. To enhance clinical interpretability, KOOS and pain evaluation (NRS) outcomes were additionally evaluated using Patient Acceptable Symptom State (PASS) and treatment failure (TF) thresholds reported by Roos [30].

Complications were classified according to the Clavien–Dindo classification adapted for orthopaedic surgery. The analysis focused on Grade 2–5 complications, as Grade 1 complications—defined as events not requiring specific treatment—are often not consistently documented in retrospective chart reviews. Grade 2 complications require pharmacological treatment beyond routine postoperative care, Grade 3 complications require surgical, endoscopic or radiological intervention, Grade 4 complications are life‐threatening events requiring intensive care management and Grade 5 represents death (Table 2) [36].

**Table 2:** Classification of adverse events according to the Clavien–Dindo classification [36].

Grade	Definition
I	No additional treatment required
II	Additional nonoperative management required
III	Additional or revision surgery required
IV	Life‐threatening complication
V	Death

### Statistical analysis

Statistical analysis was performed by an independent biostatistician.

Kaplan–Meier survival analysis was used to evaluate conversion to TKA. Continuous variables were reported as mean ± standard deviation, and normality was tested with the Kolmogorov–Smirnov test. Between‐group comparisons were performed with the Mann–Whitney *U* test for non‐normally distributed variables. Categorical variables were compared using *χ* ^2^ or Fisher's exact tests, as appropriate. The McNemar test was used for paired categorical comparisons.

Event‐time analyses considered conversion to TKA as endpoints. Survival times, event rates and survival probabilities at 5 years, at 10 years and at final follow‐up were calculated.

All tests were two‐sided, with significance set at *p* < 0.05. No correction for multiple testing was performed; results are reported in an exploratory manner. Analyses were conducted using IBM SPSS Statistics 28 (IBM Corp.).

As this was a retrospective cohort, an a priori sample size calculation was not performed. For the primary endpoint, the observed ≥10‐year non‐conversion rate (85.9%) represents a small effect compared with the predefined 80% benchmark (Cohen's *h* ≈ 0.16); with *n* = 99, post hoc power to detect such a small difference is limited. Therefore, results are presented primarily as estimates with confidence intervals (CIs) and risk‐factor analyses are interpreted as exploratory. For PROMs, standardized effect sizes (Cohen's dz) with 95% CIs were calculated to complement *p* values and MCID thresholds.

## RESULTS

### Patient demographics

**Figure 1:**
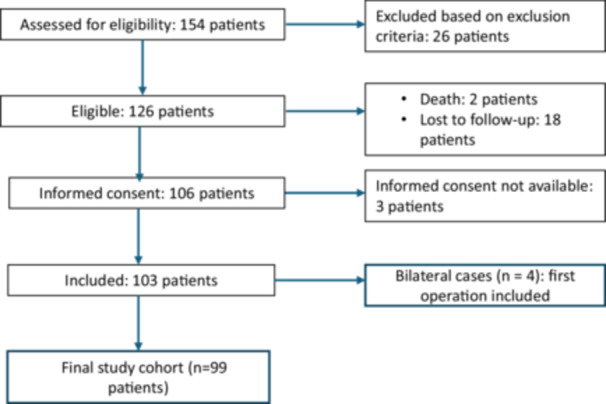
Flowchart of patient inclusion.

Of the 154 operated patients, 26 were excluded after application of the predefined inclusion and exclusion criteria, leaving 126 eligible patients. At final follow‐up, two patients had died, and 18 were lost to follow‐up. After exclusion of three patients due to incomplete informed consent and four bilateral cases (only the first operated knee included), 99 patients were available for analysis (Figure 1). The follow‐up rate was 78.6% (99/126), with a mean duration of 13.3 years (range, 10.2–18.3 years). Of these, 35 patients (35.4%) were female, and 64 (64.6%) were male. The mean age at follow‐up was 61 ± 8 years (range, 33–78), while the mean age at surgery was 48.4 ± 8.3 years (range, 23–69). Nine patients (9.1%) were smokers at the time of surgery.

Baseline characteristics available from hospital records did not differ meaningfully between patients included in the final analysis and those lost to follow‐up.

### Survival rate

**Figure 2:**
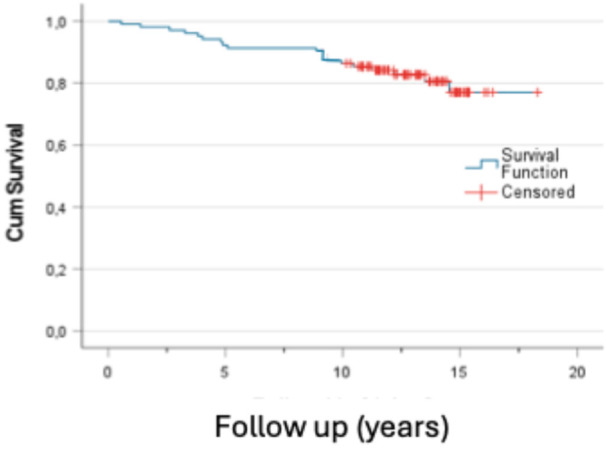
Kaplan–Meier survival curve after medial open‐wedge high tibial osteotomy. Cumulative survival is defined as freedom from conversion to total knee arthroplasty (TKA). At 5 years, survival was 91.9%, at 10 years 85.9% and at final follow‐up (mean 13.3 years) 80.8%. Crosses indicate censored cases.

Kaplan–Meier analysis demonstrated cumulative survival rates (defined as absence of conversion to TKA) of 91.9% (95% CI, 86.6–97.2) at 5 years, 85.9% (95% CI, 79.0–92.8) at 10 years and 80.8% at final follow‐up (mean 13.3 years) (Figure 2). A total of 19 patients underwent conversion to arthroplasty.

### Risk factor analysis

Patients in the conversion group were significantly older (64.9 ± 8.0 years) compared with the non‐conversion group (60.0 ± 7.6 years, *p *= 0.017). No significant associations were found for gender, smoking status, BMI, medial compartment Grade 4 cartilage damage (ICRS classification), complications or lateral compartment cartilage lesions (ICRS classification) (Table 3).

**Table 3:** Analysis for various risk factors for the conversion and non‐conversion group.

	Non‐conversion group *N* = 80	Conversion group *N* = 19	*p* Value	Statistical significance
Age at time of surgery	47.6 years ± 8.2	51.6 years ± 8.1	*p* = 0.017	significant
Rate of female patients	32.5% (26/80)	47.4% (9/19)	*p* = 0.287	n.s.
Rate of smokers	7.5% (6/80)	10.5% (2/19)	*p* = 0.647	n.s.
BMI	32.35	27.15	*p* = 0.401	n.s.
Lateral cartilage damage	12.7% (10/79)	21% (4/18)	*p* = 0.492	n.s
Femoropatellar cartilage damage	39.2% (31/79)	47% (9/18)	*p* = 0.254	n.s.
Complication rate	8.8%	15.8%	*p* = 0.399	n.s.

Abbreviations: BMI, body mass index; n.s., not significant.

### Patient satisfaction

The overall satisfaction rate was 76.8% (very satisfied 40.4%, satisfied 36.4%). Patients without conversion reported higher satisfaction (83.8% satisfied/very satisfied) compared with those with conversion (47.4% satisfied/very satisfied). This difference was statistically significant (Fisher–Freeman–Halton exact test, *p* = 0.002).

### Willingness to undergo the procedure again

At final follow‐up, 74.7% of patients indicated willingness to undergo the procedure again (52.5% ‘definitely yes’, 22.2% ‘yes’). A neutral response was given by 5.1%, while 6.1% answered ‘no’ and 14.1% ‘definitely no’, corresponding to 20.2% unwilling to repeat the surgery.

### PROMs

Functional outcomes were favourable, as reflected by KOOS subscale scores given in Table 4.

**Table 4:** Pain at rest and during walking (measured with an NRS), KOOS subscales and patient satisfaction scores.

	Non‐conversion group *N* = 80	Conversion group *N* = 19	*p* Value	Statistical significance	PASS	TF	MCID
Pain at rest	1.4 (SD ± 1.9)	1.5 (SD ± 1.9)	*p* = 0.919	n.s.	≤3	≥5	2
Walking pain	2.7 (SD ± 2.8)	2.9 (SD ± 2.4)	*p* = 0.663	n.s.	≤3–4	≥5	2
KOOS symptoms	70.0 (SD ± 21.8)	69.9 (SD ± 25.4)	*p* = 0.878	n.s.	83	56	≥10.7
KOOS pain	73.8 (SD ± 19.7)	69.6 (SD ± 25.0)	*p* = 0.698	n.s.	89	57	≥16.7
KOOS QoL	52.6 (SD ± 26.6)	42.18 (SD ± 24.1)	*p* = 0.144	n.s.	73	28	≥15.6
KOOS sport	47.9 (SD ± 29.5)	35.62 (SD ± 28.7)	*p* = 0.130	n.s.	72	28	≥12.5
Satisfaction	83.8%	47.4%	*p* = 0.002	Significant			

Abbreviations: KOOS, Knee injury and Osteoarthritis Outcome Score; MCID, minimal clinically important difference; NRS, numerical rating scale; n.s., not significant; PASS, Patient Acceptable Symptom State; SD, standard deviation; TF, treatment failure.

Pain levels at final follow‐up were low, with mean NRS scores of 1.4 at rest and 2.7 during walking, both falling below the PASS threshold of 3 points and far below the TF threshold of 5 points, indicating an acceptable symptom state in the majority of patients.

No statistically significant differences were observed between patients with and without conversion to arthroplasty in KOOS Symptoms (70.4 ± 21.8 vs. 69.9 ± 25.4; *p* = 0.878), KOOS Pain (73.8 ± 19.6 vs. 69.6 ± 25.0; *p* = 0.698), KOOS QoL (52.6 ± 26.6 vs. 42.2 ± 24.1; *p* = 0.144) or KOOS Sport (47.9 ± 29.5 vs. 35.6 ± 28.6; *p* = 0.130). When interpreted against established MCID thresholds (Symptoms ≥10.7, Pain ≥16.7, Sport ≥12.5, QoL ≥15.6), the between‐group differences did not reach clinically meaningful levels.

When compared with PASS thresholds, KOOS values in both the non‐conversion and conversion groups remained below levels associated with an acceptable symptom state across all domains. However, KOOS scores in both groups consistently exceeded TF thresholds.

### Sportive activity

The frequency of sporting activity was significantly reduced at follow‐up compared with preoperative status (McNemar test, *p* = 0.001). The mean Tegner activity score decreased from 4.7 ± 1.9 preoperatively to 3.8 ± 1.4 at follow‐up. Seventy‐nine patients reported being physically active before surgery, compared to 62 at follow‐up. Of these, 53 reported no change in activity level, 39 reported a decrease and 9 an increase.

### Complications

A total of 12 Grade 2 or 3 complications occurred, corresponding to an overall complication rate of 12.1%. The most common complication was overcorrection (*n* = 5), requiring revision osteotomy with new osteosynthesis. Non‐union occurred in two patients, one of whom was a smoker; both required revision with bone grafting. Arthrofibrosis was observed in two cases and successfully treated with arthroscopic arthrolysis. One patient required surgical evacuation of a postoperative haematoma, and one developed postoperative lymphedema managed with compression stockings. Two complications were detected in the early postoperative period (within 2 weeks postoperatively). All other complications were detected in the late postoperative period.

## DISCUSSION

The present study demonstrates a sustained TKA‐free interval following medial open‐wedge HTO using angular‐stable fixation. The 10‐year survival rate was 85.9%, and 80.8% of knees remained free from arthroplasty at a mean follow‐up of 13.3 years. These results are consistent with previous long‐term studies using the TomoFix system, which reported 10‐year survival rates of 81% [33], 84% [8], 87% [3] and 95% [17]. In contrast, Mittienen et al. reported a lower survival rate of 67% using a non‐locking steel plate implant (Arthrex) [25], underscoring the biomechanical advantage of modern angular‐stable fixation.

Patients who underwent conversion were significantly older than those without conversion. This finding is consistent with earlier reports identifying age as a key predictor of failure [15, 20, 32]. Increasing age is associated with a higher prevalence of osteoarthritis [19] and a greater likelihood of reaching the typical age range for arthroplasty. Consequently, older patients may enter the ‘TKA age window’ earlier, contributing to higher conversion rates independent of osteotomy‐related factors.

Female sex and PF cartilage damage were more common in the conversion group but did not reach statistical significance. Keenan et al. identified female sex as a significant predictor of early conversion to TKA [21]; however, their lower overall survival rate likely provided greater statistical power to detect such associations. Regarding PF degeneration, our findings align with Goshima et al., who demonstrated that PF osteoarthritis does not necessarily compromise long‐term outcomes after HTO [14].

No significant associations were found for smoking, BMI or cartilage damage in the medial or lateral compartments. The literature regarding these factors remains controversial. Obesity has been identified as a negative prognostic factor in several series [8, 16, 17], whereas other studies failed to demonstrate an influence on HTO success [12, 13]. Similarly, advanced cartilage loss has been associated with inferior outcomes in some reports [4], while Floerkemeier et al. were unable to confirm such an effect [12, 13]. Smoking has been linked to impaired bone healing and complications such as non‐union and infection [35], yet its long‐term influence on survivorship remains unclear [13, 16]. Taken together, these findings suggest that smoking may primarily affect early bone healing rather than long‐term osteotomy survival.

Interpretation against PASS and TF thresholds suggests that MOWHTO maintains patients above levels associated with TF but does not consistently restore knee function to a fully acceptable symptom state, particularly in the Sport and QoL domains [30].

Overall satisfaction reached 76.8%, comparable to previously reported rates of 70%–85% in large HTO series [12, 13]. Patients without conversion reported a satisfaction rate of 83.8%, which is similar to satisfaction levels after primary TKA of approximately 80%–85% [5]. In contrast, satisfaction was significantly lower among patients who later required arthroplasty (47.4%), although no statistically significant differences in the KOOS subdomains were observed between patients with and without later conversion to arthroplasty. This discrepancy may indicate that the need for additional surgery negatively influences subjective perception, despite comparable functional outcomes.

In our cohort, 74.7% of patients stated that they would undergo the procedure again, which is consistent with previously reported willingness rates after HTO. Floerkemeier et al. reported that 79% of patients would choose HTO again [12], and Bode et al. observed a rate of 73% [3]. These findings suggest that most patients perceive the long‐term benefits of HTO to outweigh its risks.

Sports participation declined significantly after HTO, both in frequency and in Tegner activity score. Nevertheless, most patients remained physically active at follow‐up. This is consistent with reports by Salzmann et al. and Ekhtiari et al., who showed that HTO enables continued participation in low‐impact activities, although overall activity levels often decrease compared with preoperative status [10, 31].

The overall complication rate of 12.1% was comparable to other contemporary HTO cohorts [1, 12, 35, 37, 38]. Overcorrection was the most frequent complication, reflecting known technical challenges [23]. Non‐union was rare and successfully revised, consistent with prior reports identifying it as an uncommon but recognized complication [12, 35]. Arthrofibrosis occurred in two cases and was effectively treated with arthroscopic arthrolysis [11]. Minor complications such as haematoma and lymphedema were infrequent and without long‐term sequelae.

Future improvements in HTO outcomes may arise from advances in perioperative blood management, digital planning and patient‐specific instrumentation (PSI) [2, 18, 28, 29]. In addition, increasing recognition of deformity location with digital planning tools supports more individualized surgical strategies, including femoral or double‐level osteotomy when indicated [26].

This study has some limitations. Its retrospective design introduces potential selection bias, and although follow‐up was relatively high, attrition may affect survival estimates. The sample size may have limited detection of less frequent risk factors. Furthermore, results from a single high‐volume centre may not be fully generalizable. Radiographic progression and biomechanical parameters were not systematically evaluated. A further limitation relates to the availability of detailed baseline deformity characteristics. Given the historical timeframe of this cohort (2003–2008), parameters such as deformity origin or joint line obliquity were not routinely documented and could not be retrospectively reconstructed in a reliable manner. At that time, nearly all varus deformities were corrected at the tibial level, and alignment concepts were less differentiated than in contemporary practice.

Due to the retrospective design and the historical inclusion period (2003–2008), standardized preoperative KOOS baseline data were not consistently available, precluding analysis of longitudinal treatment effects based on change scores; however, this limitation was partly mitigated by incorporating established PASS, TF and MCID thresholds to support clinical interpretation of the postoperative outcomes.

Although the follow‐up rate of 78.6% is acceptable for long‐term survivorship studies, the loss to follow‐up of 21.4% may introduce attrition bias. However, comparison of available baseline characteristics between included patients and those lost to follow‐up did not reveal relevant differences, suggesting that the analysed cohort remained representative of the original population.

## CONCLUSION

MOWHTO with angular‐stable fixation demonstrates durable long‐term joint preservation, providing high non‐conversion rates, sustained pain relief and favourable patient satisfaction. Patients in the conversion group were significantly older than those in the non‐conversion group, whereas sex, BMI, smoking status and cartilage damage were not associated with failure. These findings suggest that HTO remains a safe and effective joint‐preserving treatment option in appropriately selected patients with varus malalignment and medial compartment osteoarthritis.

## AUTHOR CONTRIBUTIONS

**Joachim‐Artus Junghans**: Data collection; coordination of statistical analysis; manuscript drafting. **Wolf Petersen**: Study conception; supervision; data interpretation; manuscript revision. **Martin Häner**: Data verification; language editing. **Bernd Wolfarth**: Scientific input; interpretation. All authors read and approved the final manuscript.

## FUNDING INFORMATION

The authors have no funding to report.

## CONFLICT OF INTEREST STATEMENT

Wolf Petersen reports consulting activities for OPED GmbH, Arthrex GmbH, Karl Storz GmbH, Stryker GmbH and Geistlich GmbH. The remaining authors declare no conflict of interest.

## ETHICS STATEMENT

The study was approved by the Medical Ethics Committee of the Medical Faculty of Charité—Universitätsmedizin Berlin (EA4/004/20) and conducted at the Department of Orthopaedics and Trauma Surgery, Martin Luther Hospital, Berlin, Germany. The study protocol was registered in the German Clinical Trials Register (DRKS00037298). All procedures performed in studies involving human participants were in accordance with the ethical standards of the institutional and/or national research committee and with the 1964 Helsinki Declaration and its later amendments. Written informed consent was obtained from all individual participants included in the study.

## Data Availability

The data supporting the findings of this study are available from the corresponding authors upon reasonable request.
